# Self-gated 4D whole-heart imaging

**DOI:** 10.1186/1532-429X-16-S1-W24

**Published:** 2014-01-16

**Authors:** Jianing Pang, Debiao Li

**Affiliations:** 1 Radiology and Biomedical Engineering, Northwestern University, Chicago, Illinois, USA; 2 Biomedical Imaging Research Institute, Cedars-Sinai Medical Center, Los Angeles, California, USA; 3 Department of Bioengineering, University of California, Los Angeles, California, USA

## Background

Suppressing cardiac and respiratory motion artifacts are major challenges in cardiac MRI. The conventional methods of ECG and diaphragm navigator gating require tedious setup, reduce the imaging efficiency significantly, are susceptible to drifts in heart rate or respiratory pattern, and can be unreliable at higher field strengths [1]. In this work, we propose a fully self-gated 4D imaging scheme with continuous 3D radial acquisition and retrospective cardiac and respiratory motion detection to overcome these limitations.

## Methods

For data acquisition, we used a contrast-enhanced, ungated spoiled gradient-echo sequence with the following sequence parameters: TR/TE = 5.5/3.0 ms, flip angle = 15 degrees, water excitation hard pulse, FOV = (400 mm)^3^, matrix = (384)^3^, and a 3D radial trajectory with 2D golden means [2]. Superior-inferior (SI) projections were inserted every 15 imaging lines as self-gating (SG) signal. A total of 100,000 projections were continuously collected, corresponding to a fixed 10 min scan time. For the subsequent off-line reconstruction, we firstly perform principal component analysis on the SG profiles to detect cardiac and respiratory motion, and the cardiac triggers were found using peak detection; secondly, we reject the arrhythmic heartbeats and assign data to different cardiac and respiratory bins; thirdly, we perform respiratory motion correction separately for each cardiac phase to combine the respiratory bins [3], and then reconstruct each cardiac phase using a self-calibrating CG-SENSE method [4].

## Results

**Figure 1:**
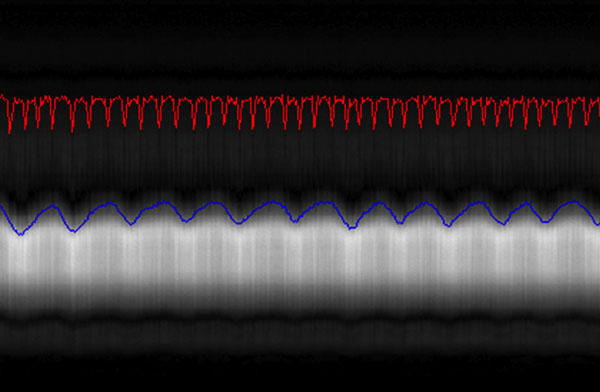
**The detected cardiac (red) and respiratory (blue) motion show good correspondence with the SG profile**.

**Figure 2:**
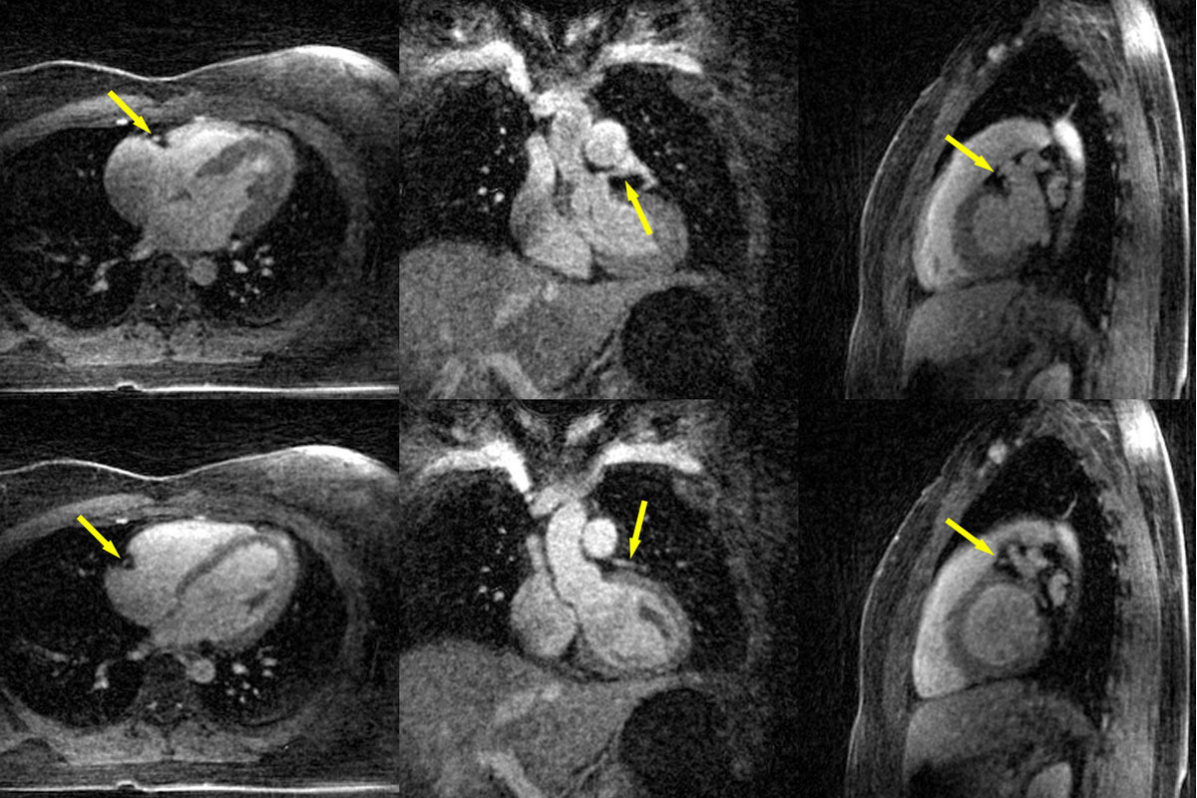
**The reconstructed mid-systole (top row) and mid-diastole (bottom row) images in axial (left), coronal (middle) and sagittal (right) orientations**. Coronary arteries are pointed out with arrows.

Figure 1 shows typical self-navigation profiles, which shows intensity variation due to cardiac motion on the top and respiratory motion on the bottom. The cardiac (red) and respiratory (blue) motion signals detected from SG display good visual correspondence with these variations. Figure 2 shows the mid-systole and mid-diastole phases out of the 16 reconstructed cardiac phases in axial, coronal and sagittal orientations with a temporal resolution of 50 ms. Coronary arteries were visible in the images.

## Conclusions

We have demonstrated a fully self-gated 4D whole-heart imaging technique with high isotropic spatial resolution and near 100% imaging efficiency through respiratory motion correction and retrospective cardiac gating. The golden 3D radial trajectory allows one to freely trade between image quality and temporal resolution, and one may determine the precise quiescent period retrospectively, which can span one or more reconstructed cardiac phases, and select the appropriate subset of data to reconstruct a high-resolution motion-free image. Future investigations are warranted to further optimize the reconstruction and sequence parameters, as well as to compare the proposed method with the conventional prospectively gated protocol.

## Funding

NIH Grant Numbers: HL38698, EB002623.
